# A new species of *Baenothrips* Crawford from China (Thysanoptera, Phlaeothripidae)

**DOI:** 10.3897/zookeys.636.10706

**Published:** 2016-11-24

**Authors:** Chao Zhao, Xiaoli Tong

**Affiliations:** 1 Department of Entomology, College of Agriculture, South China Agricultural University, Guangzhou 510642, China

**Keywords:** Baenothrips, China, fungus-feeding thrips, new species

## Abstract

A new urothripine species, *Baenothrips cuneatus* **sp. n.**, is described from China. This is distinguished from its congeners by the following combination of characteristics: dorsal surface of head having a wedge-shaped reticulation extending from median to the posterior margin; antennal segments VII–VIII is closely joined with a complete suture; the mesoacrotergite strongly constricted in the middle; abdominal tergite I divided into 5 plates; width of membranous gap between ovispan on abdominal sternite IX approximately 1/3 of the apical width of segment IX.

## Introduction

The genus *Baenothrips* Crawford currently comprises 11 species in the world, of which five are distributed in Asia (ThripsWiki 2016). These thrips are considered to be fungus-feeding, with most living in leaf litter, grass tussocks or dead twigs (Stannard 1970; Mound 1972; Okajima 1994). However, some species, such as *Baenothrips moundi* Stannard of Australia, can crawl up above soil level to grass stems, and are likely to be wind-dispersed (Mound 1972; Ulitzka and Mound 2014). The new species described below has similar dispersive behaviour, and can be collected not only in leaf litter but also on fresh leaves or stems of grass, fern, and dicotyledons. Presumably this species normally inhabits leaf litter, but crawls up fresh plants occasionally and is then dispersed by wind.

## Materials and methods

The thrips were extracted by using Tullgren funnels from leaf litter, or collected by beating vegetation over a white plastic tray using a small stick, and then sorted and preserved in 90% alcohol. Specimens were then mounted into Canada balsam on microscope slides. Structural details were examined with a ZEISS Imager A1 microscope, photos were taken by a Photometrics CoolSNAP camera, and the figures were subsequently processed with Adobe Photoshop CS6. All type specimens are deposited in the Insect Collection, South China Agricultural University (SCAU).

## Taxonomy

### Baenothrips cuneatus sp. n.

Taxon classificationAnimaliaThysanopteraPhlaeothripidae

http://zoobank.org/9CD835D4-F839-4FA7-A13E-CCCD48079809

Figs 1–4 , 5–10 , 11–14

#### Material examined

(All specimens were collected from leaf litter unless otherwise noted; females all macropterous, males all apterous).

**Holotype.** Female macroptera, **CHINA**, Guangdong province, Gaozhou County, Yuntan Town, Mt. Sanguanshan (21°55’10”N, 111°8’40”E), in leaf litter of *Acacia auriculiformis* (Fabaceae), 15.xii.2014, Chao Zhao (in SCAU).

**Paratypes.** 8 females 1 male, taken with holotype; 3 females 7 males, same locality and habitat as holotype, 5.ix.2015, Zhaohong Wang. **CHINA, Hunan**: 1 female, Yanling County, Shennong Valley (26°29'N, 114°1'E), on grass stem or leaf, 15.ix.2014, Chao Zhao. 1 female, Yanling County, Shennong Valley (26°29'N, 114°1'E), in leaf litter of *Cryptomeria fortune* (Taxodiaceae), 16.ix.2014, Chao Zhao. **Guangdong**: 1 male, Shixing County, The Chebaling National Nature Reserve (24°42'N, 114°11'E), 11.x.2002, Zhiwei Li; 3 females 1 male, Huizhou City, Mt. Nankunshan (23°38'N, 113°50'E), 11.xii.2002, Zhiwei Li; 1 female, Guangzhou City, Longdong Forest Park (23°14’ N, 113°24’ E), 5.xii.2004, 1 female, in leaf litter of *Acacia auriculiformis*, 1.xii.2006, Jun Wang; 1 female, Dongguan City, Mt. Yinpingshan (21°55’10”N, 111°8’40”E), on fresh leaf of *Stenoloma chusanum* (Lindsaeaceae), 10.ix.2014, Chao Zhao; 3 females, Guangzhou City, Mt. Maofengshan (23°17'N, 113°27'E), on fresh leaf or stem of *Dicranopteris dichotoma* (Gleicheniaceae), 4.i.2016, Chao Zhao; 3 females, Shenzhen City, Mt. Wutongshan (22°24'N, 113°17'E), on fresh leaf or stem of *Dicranopteris dichotoma*, 29.iv.2016, Chao Zhao. **Guangxi**: 1 female, Nanning City (22°48'N, 108°22'E), on fresh leaf or stem of *Pennisetum purpureum* (Poaceae), 3.x.2012 (Shulan Yang); 1 females, Shangsi County, Shiwandashan National Forest Park (25°54'N, 107°54'E), on grass stem or leaf, 25.vii.2016, Chao Zhao. **Yunnan**: 1 female, Jinghong City, 5.iv.1987, Xiaoli Tong. **Hainan**: 1 male, Baisha County, Yinggeling National Nature Reserve, Yinggezui Protection Station (18°03'N, 109°54'E), on fresh leaf of *Argyreia acuta* (Convolvulaceae), 8.i.2016, Xiaoli Tong.

#### Description.

**Figures 1–4.**
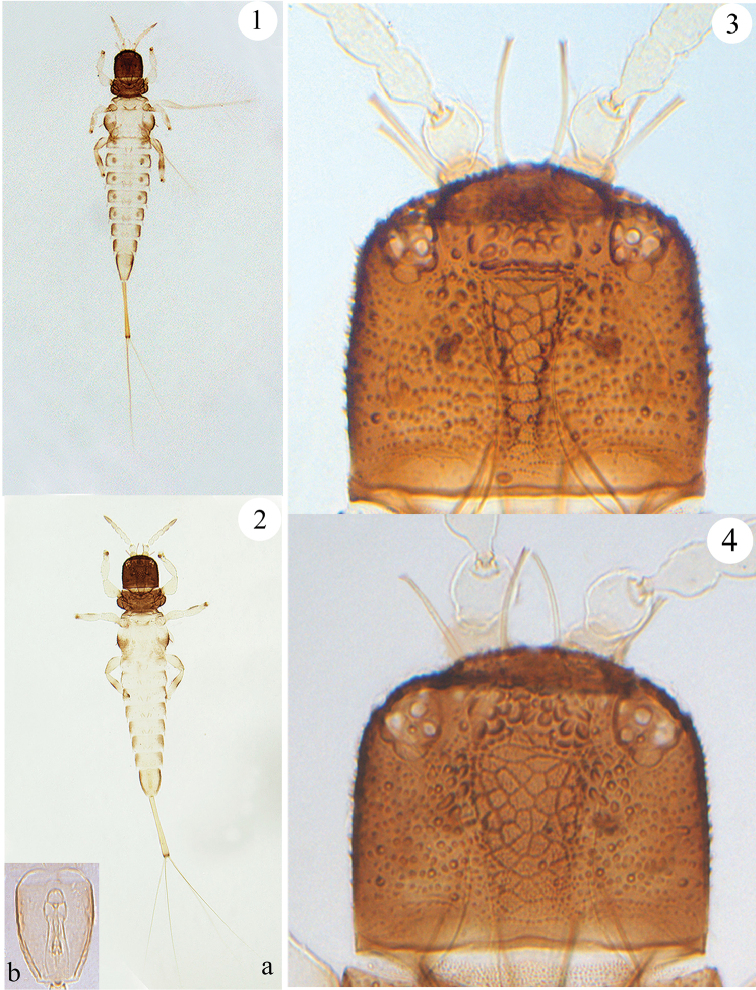
*Baenothrips cuneatus* sp. n. **1** female habitus **2a** male habitus **2b** male genitalia **3** head of female **4** head of male.

**Female macroptera** (Fig. 1): Head and prothorax dark brown; pterothorax yellowish white with dark brown anteriorly and laterally; abdominal tergites I–IX yellowish white with brown laterally, of which tergites II–V each with a pair of light brown circular patches on either side; tube yellow with extreme apex dark brown. All coxae, trochanters and apical half of tarsi brown; fore and mid femora yellowish white except for inner base brown, hind femora pale yellowish brown with brown on dorsal margins; fore tibiae yellowish white, mid tibiae yellowish yellow shaded with light brown on outer margins, hind tibiae whitish but brown medially. Antennal segment I pale brown, segments II–VI yellowish white, segments VII and VIII pale brown.

*Head* (Fig. 3) almost as long as broad or a little shorter; head broadly rounded in front, with three pairs elongate cephalic setae on anterior margin; dorsal surface tuberculate and with a wedge-shape reticulation extending from middle to posterior margin; cheeks almost straight. Eyes with approximately eight facets dorsally and six ventrally, of which three dorso-lateral facets are distinctly larger than the others; three ocelli present, anterior ocellus placed between inner cephalic setae, posterior ocelli behind outer pair of cephalic setae and placed close to eyes. Antenna 8-segmented, arising ventrally (Fig. 13), segments VII–VIII closely joined with a complete suture; segment III with no sense cones, IV with two sense cones, each approximately two-thirds as long as the segment; segment V with one sense cone, situated outside of apex; segments VI and VII each with one sense cone dorsally. Maxillary stylets retracted to base of compound eyes, approximately one-third of head width apart medially.

**Figures 5–10.**
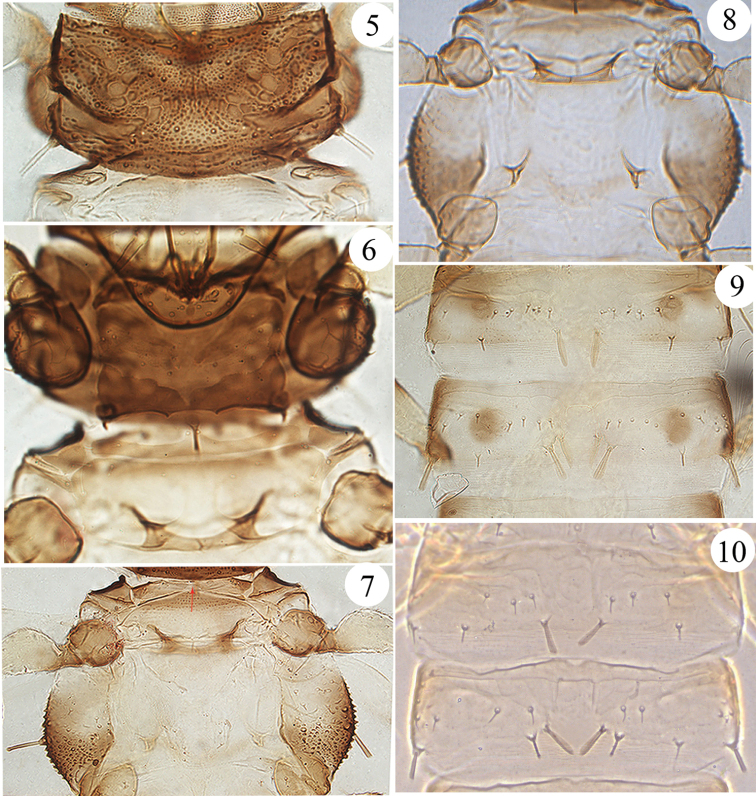
*Baenothrips cuneatus* sp. n. **5** pronotum **6** ventral view of prothorax **7** dorsal view of pterothorax (arrow indicates mesoacrotergite constricted medially) **8** ventral view of pterothorax (show meso- and metasternal furcae) **9** abdominal tergites II–III of female **10** abdominal tergites II–III of male.

**Figures 11–14.**
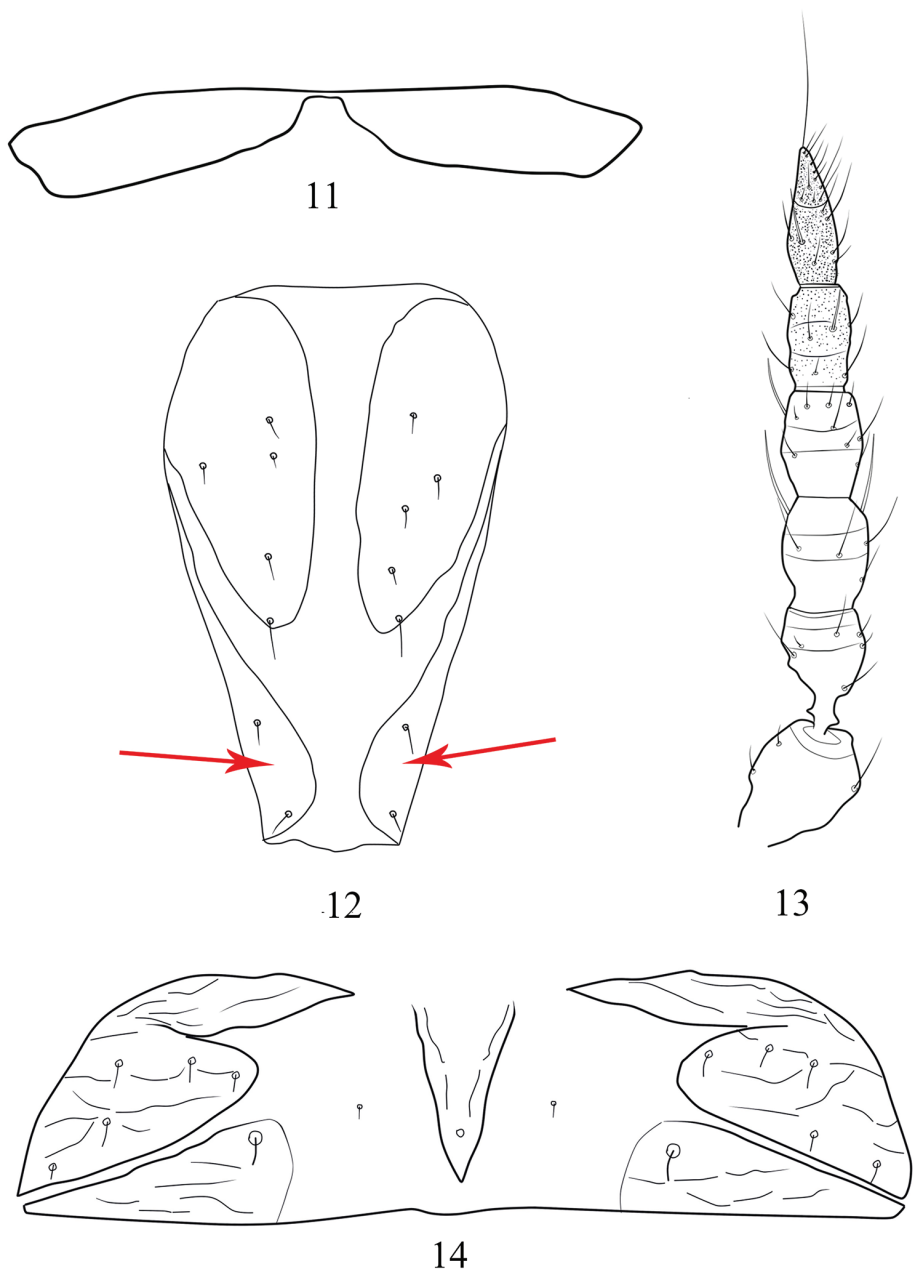
*Baenothrips cuneatus* sp. n. (female) **11** mesoacrotergite **12** abdominal sternite IX (arrow indicates the ovispan) **13** antennal segments II–VIII **14** abdominal tergite I.

*Pronotum* rectangular (Fig. 5), shorter than head, dorsal surface with irregular sculpture and wart-like tubercles; epimeral setae well developed. Mesoacrotergite strongly constricted medially by a very narrow bridge (Figs 7, 11); mesonotum sculptured with transverse dotted lines on anterior third; meta-epimeron bulging with one well developed seta. Fore wing bulging at base without basal setae; both fore wing and hind wing with a median vein or thickening, and with many, but not closely spaced, fringe cilia. Basantra weakly developed, largely membranous; ferna well developed, strongly narrowed posteromedially (Fig. 6); mesopresternum complete and transverse; mesoeusternum anterior margin entire. Mesosternal furcae fused in the middle; metasternal furcae placed laterally and widely separated (Fig. 8). All tarsi unarmed.

*Abdominal tergite* I divided into five plates, a slender median longitudinal plate bearing a campaniform sensillum (Fig. 14); tergite II with a pair of expanded wing-retaining setae; tergites III–VII with two pairs of wing-retaining setae, inner pair knife-like and outer pair fin-shaped (Fig. 9; cf. Bhatti 2002: fig 15a); tergites III–VIII posterolateral setae enlarged, each with a transverse row of 12–18 short setae medially; tergite IX 4.4 times as long as distal wide; ovispan slightly reduced, the width of membranous gap between ovispan approximately 1/3 of the apical width of segment IX (Fig. 12). Tube approximately twice as long as head with three pairs of anal setae; anal setae nearly 2.2 times as long as tube, but median dorsal pair half as long as the lateral two pairs.

**Measurements** (holotype female in microns). Body length 1680. Head length 165; maximum width 170; anterior cephalic setae, median pair 73, lateral inner pair 65, outer pair 55. Pronotum length 100; median width 185; epimeral setae 27. Metathoracic epimeral setae 30. Abdominal tergite IX length 175, basal width 100, distal width 40. Tube length 315, basal width 20, apical width 30; anal setae, dorsal pair 335, lateral pairs 750. Antennal segments I–VIII length (width) as follows: 22 (28), 30 (29), 36 (25), 33 (24), 30 (21), 28 (19), 24 (14), 22 (10).

**Male aptera** (Fig. 2a): Head with only two pairs of elongate cephalic setae on anterior margin; wedge-shape reticulation of head wider than in female (Fig. 4); abdominal tergites III–VII without brown circular patches on either side; tergites II–VII with only one pair of wing-retaining setae (Fig. 10); abdominal tergite IX nearly three times as long as distal wide. Male genitalia as in figure 2b.

**Measurements** (paratype male in microns). Body length 1180. Head length 120; maximum width 140; two pairs of anterior cephalic setae 42. Pronotum length 80; median width 165; epimeral setae 20. Metathoracic epimeral setae 18. Abdominal tergite IX length 120, basal width 80, distal width 40. Tube length 225, basal width 15, apical width 25; anal setae, median dorsal pair 230, lateral pairs 550. Antennal segments I to VIII length (width) as follows: 16 (27), 25 (27), 35 (23), 27 (23), 35 (21), 24 (19), 25 (15), 17 (13).

#### Etymology.

The specific epithet is from the Latin adjective “*cuneatus*” meaning wedge-shaped, and refers to the shape of reticulation on head.

#### Distribution.

China (Hunan, Guangdong, Guangxi, Yunnan, Hainan).

#### Remarks.

Only two species of the genus *Baenothrips* Crawford are validly recorded from China, *Baenothrips cuneatus*, and *Baenothrips ryukyuensis* Okajima. The record by Kudô (1978), of *Baenothrips asper* (Bournier) from China in Taiwan, was considered by Okajima (1994) to be a misidentification and to actually refer to *ryukyuensis*. Similarly, Bhatti (2002) suggested that the species *Baenothrips asper* is known only from Africa, and that the Asian records refer to some other species. Recently, *Baenothrips ryukyuensis* was recorded by Dang and Qiao (2014) from Fujian, China. Moreover, during sorting of specimens *Baenothrips* from China we found in our collections slide-mounted specimens labelled by Wang and Tong (2007) as *Baenothrips murphyi* (Stannard), and recognised that these actually represent *Baenothrips cuneatus*, the new species described above.

*Baenothrips cuneatus* sp. n. is most closely related to *Baenothrips asper* (Bournier) in colour pattern and several other features, but in the new species, dorsal surface of head having a wedge-shaped reticulation extending from median to the posterior margin; the mesoacrotergite is strongly constricted medially by a very narrow bridge (Figs 7, 11) as in *Baenothrips moundi* (Stannard) (cf. Bhatti 2002: fig 25) and the membranous gap (Fig. 12) between the ovispan on abdominal sternite IX is much wider than those in *Baenothrips asper* (Bournier) (cf. Bhatti 2002: fig 18). In addition, there are other five species of the genus occurring in Asia, some of them are also similar to *Baenothrips cuneatus* sp. n., but this new species can be distinguish from them by the below key.

### Key to Asian species of *Baenothrips* (female)

**Table:** 

1	Head with two pairs of prominent anterior cephalic setae	**2**
–	Head with three pairs of distinct anterior cephalic setae	**3**
2	Two pairs of anterior cephalic setae situated laterally, and median pair of anterior cephalic setae absent; macroptera	***Baenothrips quadratus***
–	Only one lateral cephalic seta on either side, and one median pair of anterior cephalic setae present; aptera	***Baenothrips indicus***
3	Antenna 8-segmented (suture between segments VII and VIII complete)	**4**
–	Antenna 7-segmented; except for epimeral setae, pronotum also having a pair of well-developed midlateral setae; macroptera or brachyptera	***Baenothrips minutus***
4	Head with a wedge-shaped reticulation extending from median to the posterior margin; the mesoacrotergite is strongly constricted medially (Figs 7, 11); abdominal tergite I divided into five plates (Fig. 14); the width of membranous gap (Fig. 12) between ovispan is approximately 1/3 of the posterior margin of abdominal sternite IX; macroptera	***Baenothrips cuneatus* sp. n.**
–	Head reticulate just medially; the mesoacrotergite is not constricted medially; abdominal tergite I entire; the membranous gap between the ovispan is reduced to a longitudinal narrow cleft; macroptera or aptera	**5**
5	Three pairs of ocelli present, lateral ocelli placed close to eyes; basantra seemingly absent; macroptera	***Baenothrips murphyi***
–	Ocelli absent; basantra weakly developed; aptera	***Baenothrips ryukyuensis***

## Supplementary Material

XML Treatment for Baenothrips cuneatus
